# Long-Term Psychological, Economic and Spiritual Consequences for Family Caregivers of Former ICU Patients

**DOI:** 10.1192/j.eurpsy.2026.11311

**Published:** 2026-07-31

**Authors:** F. Tatsis, M. Gouva, E. Dragioti, S. Georgakis, F. Veroniki, K. Stamatis, G. Papathanakos, V. Koulouras

**Affiliations:** 1 Intensive Care Unit, University Hospital of Ioannina; 2 Scientific Laboratory of Psychology and Person-Centered Care, Department of Nursing, University of Ioannina, Ioannina, Greece

## Abstract

**Introduction:**

Family caregivers of patients in the Intensive Care Unit (ICU) face a complex burden. This includes the emotional distress of witnessing a loved one’s critical illness and significant disruptions to their professional and financial stability, especially during prolonged hospitalizations. While existing research documents aspects of caregiver distress, holistic studies examining the intersection of psychological, economic and spiritual dimensions remain scarce. There is a clear need for long-term, integrative investigations to understand the evolving impact of caregiving and to inform targeted support strategies.

**Objectives:**

This study aimed to explore the long-term psychological, economic, occupational and spiritual consequences experienced by family caregivers of former ICU patients. Specifically, it sought to identify key predictors of psychological and spiritual well-being and to uncover distinct caregiver profiles based on patterns of distress, coping and adaptation.

**Methods:**

This cross-sectional follow-up study assessed the long-term impact on 92 family members, six years after a relative’s ICU admission. We examined economic hardship, job disruption, psychological distress and spiritual coping using validated tools (e.g., SCL-90-R, SpREUK, FCOPES). Multivariate regression identified predictors of psychological and spiritual adjustment, while cluster analysis revealed distinct caregiver subgroups.

**Results:**

The findings showed that job loss was a strong predictor of anxiety and irritability, while reduced working hours appeared to protect against emotional distress. Financial strain was less strongly associated with mental health. Spirituality had a dual role: trust and reflection supported coping, but deeper spiritual involvement was sometimes linked to higher depression. Traits like resilience, forgiveness and cognitive flexibility were linked to lower distress, while neuroticism increased vulnerability. Cluster analysis identified three caregiver profiles: one with high emotional and financial burden, one with mixed psychological and spiritual responses and one with low distress across all domains.

**Conclusions:**

The findings underscore that economic hardship is a central, not incidental, factor in caregiver distress. While spirituality can offer meaningful support, it may also reflect underlying emotional struggle. Interventions should be multidimensional -combining financial assistance, targeted psychological care and culturally sensitive spiritual support- to effectively address the complex needs of family caregivers following intensive care experiences.

**Disclosure of Interest:**

None Declared

